# PAX8: a sensitive and specific marker to identify cancer cells of ovarian origin for patients prior to neoadjuvant chemotherapy

**DOI:** 10.1186/1756-8722-6-60

**Published:** 2013-08-19

**Authors:** Yue Wang, Yiying Wang, Jie Li, Zeng Yuan, Bingbing Yuan, Tingguo Zhang, Janiel M Cragun, Beihua Kong, Wenxin Zheng

**Affiliations:** 1 Department of Obstetrics and Gynecology, Henan Province People’s Hospital, Zhengzhou, Henan 450003, China; 2 Department of Pathology, University of Arizona College of Medicine, Tucson, AZ 85724, USA; 3 Department of Obstetrics and Gynecology, Qilu Hospital, Shandong University, Ji’nan, Shandong 250012, China; 4 Whitehead Institute for Biomedical Research, Cambridge, MA 02142, USA; 5 Department of Pathology, Qilu Hospital, Shandong University, Ji’nan, Shandong 250012, China; 6 Department of Obstetrics and Gynecology, College of Medicine, University of Arizona, 1501 N. Campbell Avenue, #5205, Tucson, AZ 85724, USA; 7 Arizona Cancer Center, University of Arizona, Tucson, AZ 85724, USA

**Keywords:** PAX8, Ascitic fluid, Ovarian cancer, Neoadjuvant chemotherapy, Origin, Marker

## Abstract

**Background:**

Neoadjuvant chemotherapy followed by cytoreduction surgery has been used where an accurate cytologic or pathologic diagnosis is usually required before the initiation of neoadjuvant chemotherapy. However, it is difficult to make definitive diagnosis of presence of cancer cells, particularly gynecologic versus non-gynecologic origin, from those ascites specimens due to the absence of specific biomarkers of gynecologic cancers. In the present study, we evaluated if, in addition to the routine morphologic diagnosis, the biomarker PAX8 could be useful in recognition of ovarian epithelial cancer cells prior to the neoadjuvant chemotherapy.

**Methods:**

Two hundred and two cytology specimens including 120 pretreatment ovarian cancer samples, 60 benign controls, and 22 malignant non-gynecologic cases were studied. All cytology slides were morphologically reviewed in a blinded fashion without knowing corresponding pathology diagnosis, if present. A total of 168 cytology specimens with a cell block were stained with PAX8 and Calretinin. These included patients with potential for ovarian cancer neoadjuvant chemotherapy (n = 96), metastatic cancers (n = 22), and benign controls (n = 50).

**Results:**

Among the 96 ascitic samples prior to neoadjuvant chemotherapy, 76 (79%) showing morphologic features consistent with cancers of ovarian primary were all PAX+/Calretinin-. The remaining 20 (21%) cases were positive for adenocarcinoma, but morphologically unable to be further classified. Among the 22 metastatic cancers into the pelvis, one case with PAX8+/Calretinin- represented a renal cell carcinoma and the remaining 21 PAX8-/Calretinin- metastatic cancers were either breast metastasis (n = 4) and the metastasis from gastrointestinal tract (n = 17). Among the 50 benign control pelvic washing cases, 5 PAX8+/Calretinin-cases represented endosalpingiosis (n = 4) and endometriosis (n = 1), 25 PAX8-/Calretinin + cases showed reactive mesothelial cells, and the remaining 20 specimens with PAX8-/Calretinin- phenotype typically contained inflammatory or blood cells without noticeable diagnostic epithelia.

**Conclusions:**

PAX8 identifies all Müllerian derived benign or malignant epithelia. When combining with Calretinin, PAX8 is a sensitive marker to diagnose the carcinomas of ovarian origin, which will be ideal to be used for those patients with a possible advanced ovarian cancer prior to receiving neoadjuvant chemotherapy.

## Introduction

Primary tumor debulking (cytoreduction) followed by chemotherapy is considered the standard of care for patients with advanced stage of either tubal/ovarian or primary peritoneal cancers. As an alternative to this practice, neoadjuvant chemotherapy followed by cytoreduction surgery has been used where an accurate cytologic or pathologic diagnosis is usually required before the initiation of neoadjuvant chemotherapy [1,2]. In addition, obtaining a pelvic washing sample is a common surgical procedure for gynecologic malignancies. The pathologic findings from those washing specimens also have a significant impact for the decision of clinical management.

However, it is well known that cytologists/pathologists feel difficult to make definitive diagnosis of presence of cancer cells, particularly gynecologic versus non-gynecologic origin, from those pre-surgical ascites or pelvic washing specimens. This is partially related to absence of specific biomarkers of gynecologic cancers [3,4].

Therefore, finding a marker with high sensitivity and specificity to be added to the traditional immunohistochemistry (IHC) panel of antibodies is needed. PAX8 is a member of the pair-box (PAX) family of transcription factor genes. Recent studies showed that PAX8 is a useful marker to distinguish gynecologic cancers from non-gynecologic malignancies including malignant mesotheliomas, cancers of gastrointestinal origin and breast cancers, which are common confusion sources in clinicopathological practice [5-7]. In this study, we evaluated the utility of PAX8 antibody in recognition of cancer cells of ovarian origin for the patients prior to receiving neoadjuvant therapy.

## Methods

### Case selection

A total of 202 cytology specimens including 120 pretreatment ovarian cancer samples, 60 benign controls, and 22 malignant non-gynecologic cases were studied. The 120 patients were treated with neoadjuvant chemotherapy for an apparent advanced stage ovarian cancer between 1979 and 2010. These included cases from our previous study [1] (n = 60), University of Arizona (n = 30) and Shandong University, China (n = 30). The benign control cases were from pelvic washing specimens of patients with benign gynecologic diseases (leimyomata, endometriosis, and benign ovarian tumors). The malignant non-gynecologic cancer controls included colorectal cancer (n = 9), breast cancer (n = 8), renal cell carcinoma (n = 3), pancreatic cancer (n = 2). The ovarian cancer patients had the following clinicopathologic features. Ninety of the 120 (75%) patients had evidence of extra-abdominal tumor spread prior to treatment, 110 had ascites, 35 had unilateral pleural effusion and 16 had bilateral pleural effusions. 102 patients with known preoperative serum CA125 levels showed values > 500 U/ml, 67 being > 1500 U/ml. Patients’ characteristics are summarized in Table 1. The study was performed with the approval by corresponding institutional review board.

**Table 1:** Patient characteristics

**Site of cancer**	
Intra-abdominal	30
Extra-abdominal	90
Ascites	
Yes	110
No	10
Pleural Effusions	
None	69
Unilateral	35
Bilateral	16
Preoperative Serum CA125(U/ml)	
<500	18
501-1500	35
>1500	67

### Ascites sample preparation

Ascitic fluid was tapped from candidates with potential neoadjuvant chemotherapy. An average of 50 to 100 ml of ascites was obtained. The samples were treated with Cell-Lite to lyse red blood cells and then spun down in 50-ml sterile plastic tubes. At least one slide with Papanicolaou staining was made from each sample for cytologic examination. A cell block was also made when a cell pellet was visible.

### Cytologic evaluation

All pretreatment or corresponding cytology slides were reviewed in a blinded fashion without knowing corresponding pathology diagnosis. They were characterized as benign, atypical or suspicious for malignancy, or malignant. In malignant category, the cases were further divided into consistent with ovarian epithelial cancer (OEC) or cytologically non-classifiable. The following criteria were used to identify the presence of malignant cells consistent with ovarian primary based on well-established cytologic features published previously [1,8,9]: Malignant cells with abundant non-mucinous cytoplasm suggested serous carcinoma; Papillary structures with the above cytologic features consistent with serous carcinoma, particularly when psammoma bodies were seen; Malignant cells with vacuoles or a hint of clear cell differentiation suggested clear cell carcinoma; The presence of prominent nucleoli was looked for as they frequently were present in serous and clear cell carcinomas; When endocervical type malignant cells were present, they were most compatible with a Müllerian or ovarian primary.

The presence of endosalpingiosis and psammomatous microcalcifications was also noted using previously published criteria [8,10].

### Immunohistochemistry

Among the 202 samples, a total of 168 cytology specimens with a cell block were stained with PAX8 and Calretinin. These included patients with potential for ovarian cancer neoadjuvant chenotherapy (n = 96), metastatic cancers (n = 22), and benign controls (n = 50). IHC was carried out using the Envision Plus/Horseradish Peroxidase system (Dako, Carpinteria, CA), a polyclonal antibody to PAX8 (Proteintech Group Inc, Chicago, IL, 1:800 dilution), all cases stained at a dilution of 1:300. Calretinin, a routine antibody used in surgical pathology practice, has been described elsewhere [11]. In brief, sections from paraffin-embedded cell blocks were incubated in hydrogen peroxidase and absolute alcohol for 30 minutes to block endogenous peroxidase activity. Antigen retrieval was carried out using pressure cooker pretreatment in citrate buffer (pH = 6.0). Tissue sections were subsequently incubated with the primary antibody for 40 minutes at room temperature. After TBS rinses, the tissue was incubated using the Envision Plus secondary antibody for 30 minutes followed by diaminobenzidine for 5 minutes. Appropriate positive (an ovarian cancer known to be positive for PAX8 and a mesothelioma known to be positive for Calretinin) and negative (incubation with secondary antibody only) controls were stained in parallel for each round of IHC.

PAX8 and Calretinin were evaluated for nuclear staining. Immunoreactivity was graded based on the stainings in target cells by comparing the H&E morphology. Positive staining was defined as equal or more than 50% of the target cells showing intense nuclear immunoreaction, while negative if less than 50% of target cells were stained or the staining intensity was weak or moderate. The intensity staining was referenced by comparing the positive controls.

## Results

### Microscopic diagnosis for cases with potential neoadjuvant chemotherapy

**Figure 1:**
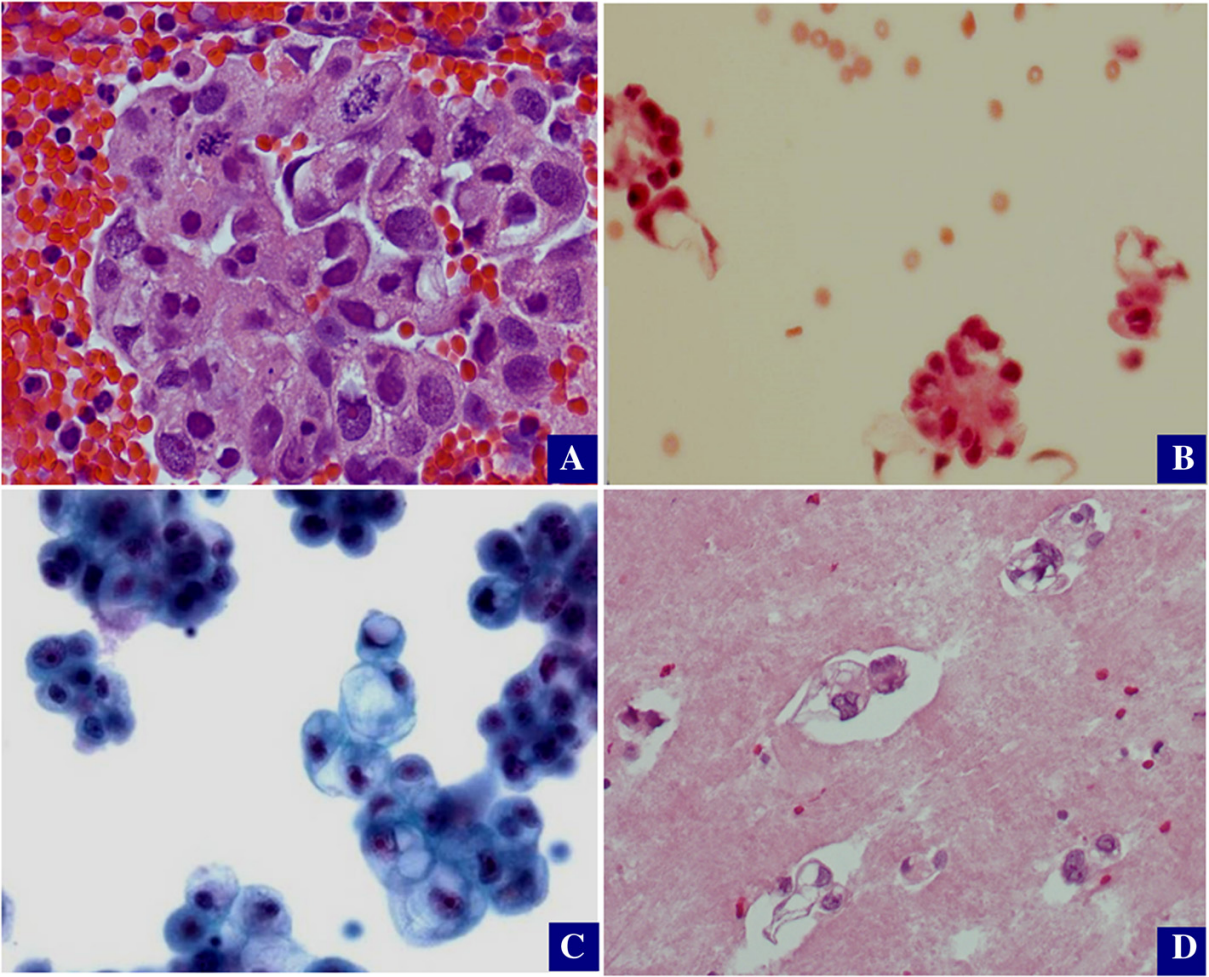
**Cytologic features of ovarian epithelial cancer.** Ovarian epithelial cancers typically contain abundant non-mucinous cytoplasm in serous carcinoma **(A)**; Papillary structures **(B and C)** with the above cytologic features are commonly seen in either serous or clear cell carcinoma; Cancer cells with vacuoles or a hint of clear cell differentiation are suggestive of clear cell carcinoma **(C and D)**.

Among the 120 ascites samples from patients who might receive neoadjuvant chemotherapy, 96 cases with a cytology block available for further analysis. The cytology slides from these 96 cases were reviewed under microscope based on the diagnostic criteria described above. The following results were obtained: adenocarcinoma, consistent with OEC (n = 76, 79%) and positive for adenocarcinoma with uncertain primary (n = 20, 21%). Representative pictures for cancers consistent with ovarian primary are illustrated in Figure 1.

### PAX8 and Calretinin staining results

**Figure 2:**
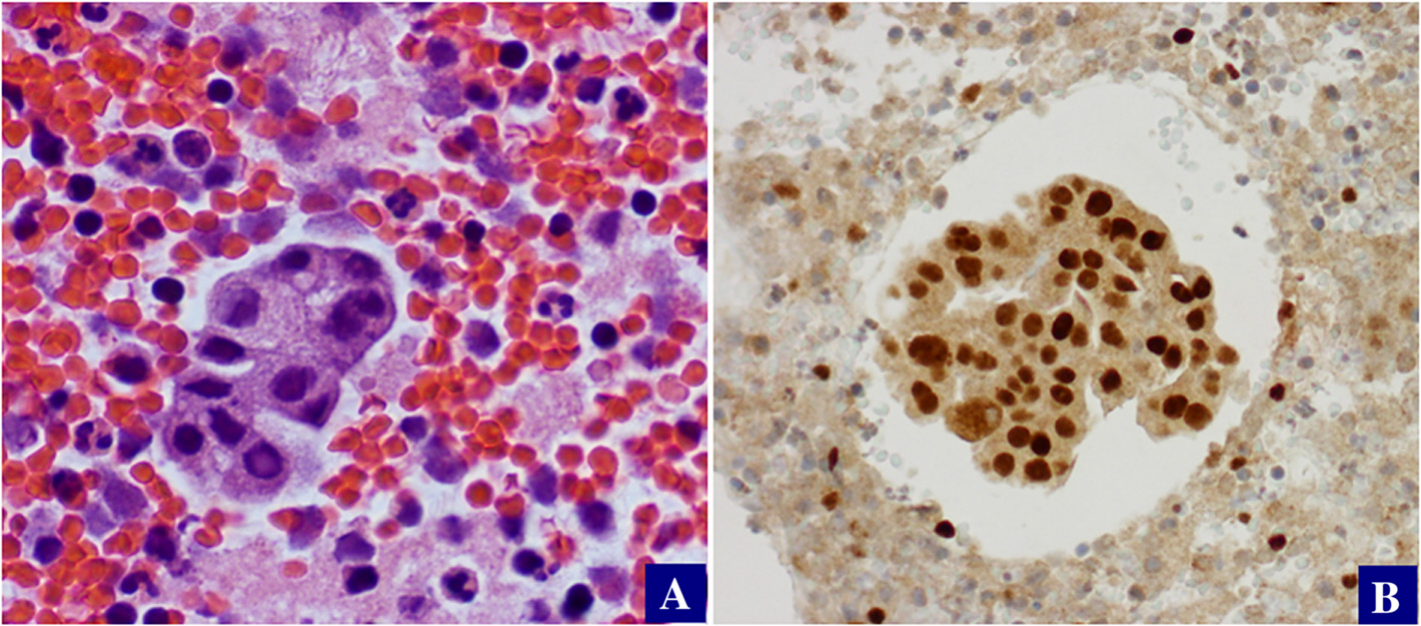
**PAX8 staining in high-grade serous carcinoma.** Ovarian high-grade serous carcinoma **(A)** typically shows diffuse positive staining for PAX8 **(B)**.

**Figure 3:**
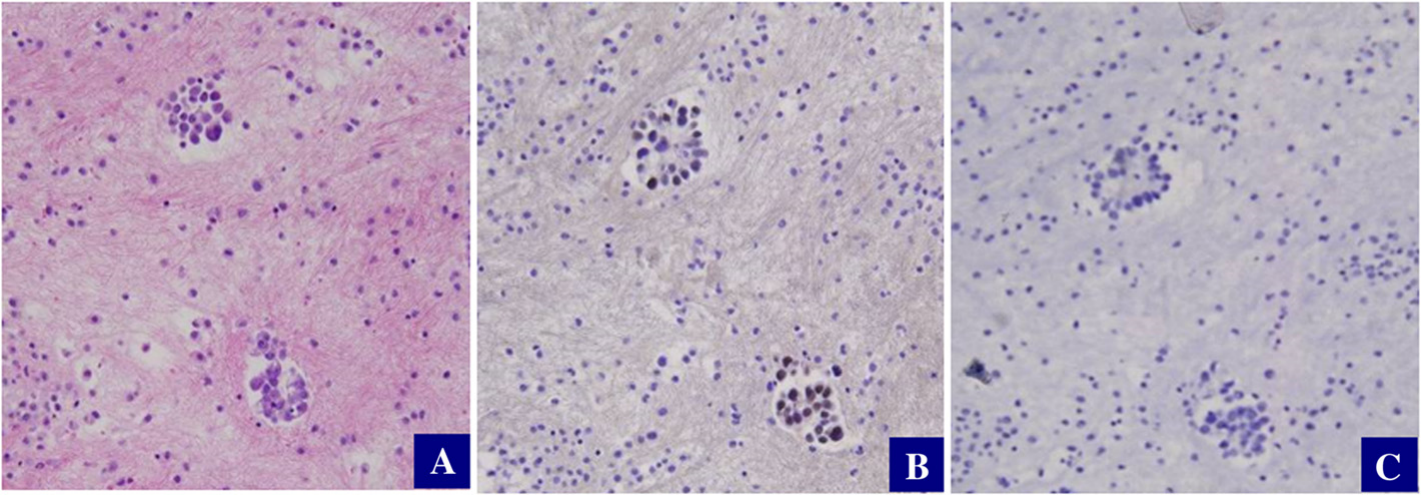
**PAX8 and Calretinin staining in low-grade serous carcinoma.** Ovarian low-grade serous carcinoma **(A)** shows positive staining for PAX8 **(B)**, but negative for Calretinin **(C)**.

The 76 cases showing morphologic features consistent with cancers of ovarian primary were all positive for PAX8 and negative for Calretinin. Among the 20 cases with uncertain primary organ sites, the results were as follows: PAX8+/Calretinin- (n = 13), PAX8-/Calretinin + (n = 2), and PAX8-/Calretinin- (n = 5). Representative pictures are presented in Figures 2 and 3.

Among the 22 meatstatic cancers into the pelvis, the staining results were as follows: PAX8+/Calretinin- (n = 1), PAX8-/Calretinin + (n = 0), and PAX8-/Calretinin- (n = 21).

Among the 50 cases with benign gynecologic diseases, the staining results showed: PAX8+/Calretinin- (n = 5), PAX8-/Calretinin + (n = 25), and PAX8-/Calretinin- (n = 20).

### Clinicopathologic correlations between immunostaining results and permanent pathologic findings

Among the 76 cases showing positive PAX8 and negative Calretinin, they were 100% correlated to clinicopathologic diagnosis of OEC. Among the 20 uncertain cases, 13 (65%) were confirmed with OEC both immunohistochemically (PAX8+/Calretinin-) and clinicopathologically. The 2 cases showing PAX8-/Calretinin + turned out to be malignant mesothelioma. The 5 patients showing PAX8-/Calretinin- phenotype represented 2 metastatic breast, 1 colon, 1 gastric, and 1 pancreatic cancer.

Among the 22 metastatic cancers in a cancer control group, one showing PAX8+/Calretinin- represented a renal cell carcinoma, which is known to have such phenotype [12-14]. The 21 PAX8-/Calretinin- metastatic cancers were either breast metastasis (n = 4) and the metastasis from gastrointestinal tract (n = 17).

We included 50 benign control cases in the study. The 5 PAX8+/Calretinin-cases in this category represented endosalpingiosis (n = 4) and endometriosis (n = 1). The cases with PAX8-/Calretinin + represented reactive mesothelial cells within the pelvic washing specimens. Specimens with PAX8-/Calretinin- phenotype typically contained inflammatory or blood cells without much diagnostic epithelia. The above results are summarized in Table 2.

**Table 2:** Summary of the PAX8 and Calretinin staining results in studied cases and controls

	**#Cases**	**PAX8+/Cal (%)**	**PAX8+/Cal+ (%)**	**PAX8-/Cal (%)**	**PAX8-/Cal+ (%)**
**1**^**0 **^**OvCa**	76	76 (100)	0 (0)	0 (0)	0 (0)
**Uncert 1**^**0**^	20	13 (65)	0 (0)	5 (25)	2 (10)
**Metastatic**	22	1 (5)	0 (0)	21 (95)	0 (0)
**Benign**	50	5 (10)	0 (0)	20 (40)	25 (50)

Cal: Calretinin; +: positive; -: negative; 1^0^: primary; OvCa: ovarian cancer; Uncert: uncertain.

## Discussion

Ovarian cancer is the eighth most common cancer among women with a lifetime risk of about 1 in 70 [15]. It accounts for approximately 30% of all cancer deaths within the female genital tract and is the most aggressive cancer compared with its incidence rate [16].

The peritoneal cavity is a common site of involvement for various reactive, inflammatory, and neoplastic processes. Metastases from primary ovarian malignancies are particularly common in this location. Approximately 70% of OEC, the most common form of ovarian cancers, are not diagnosed until the disease has involved the upper abdomen or spread beyond the abdominal cavity [17]. Traditional strategy for the management of advanced ovarian cancer is primary cytoreductive surgery with intraoperative peritoneal washing cytology followed by adjuvant chemotherapy. However, it seems that such strategy does not increase overall patient survival significantly. Neoadjuvant chemotherapy (NACT) is emerging as an effective treatment modality in many locally advanced solid tumors, including breast, gastrointestinal and bone and soft tissue malignancies these years. The rationale behind NACT protocol is to make inoperable advanced disease operable, to increase cancer resection rates, and to facilitate potential organ conservation [18], if applicable. Nowadays, NACT has been advocated by NCCN guideline for patients with advanced ovarian cancer with an aim to improve tumor debulking and overall survival.

Approximately 70-80% advanced ovarian cancers could be accurately diagnosed based on clinicopathological and imaging studies. However, the remaining 20-30% of ovarian cancers may cause clinical management problem due to a similar clinicopathological presentations. Therefore, it is important to have cytologic or pathologic diagnosis for those patients with probable ovarian cancers prior to NACT. But concerns have been raised about the reliability of using cytology to diagnose ovarian cancer since non-gynecologic cancer cells can not be reliably differentiated under microscope. In 2003, Schwartz and Zheng reported the role of cytology in pretreatment diagnosis of ovarian cancer followed by NACT and recommended that it was essential for the clinician and the cytologist or pathologist to communicate with each other to make a better diagnosis by using cytological material [1]. The bottleneck limiting the efficient application of pre-neoadjuvant cytology is lack of sensitive and effective biomarkers for ovarian cancer in this setting.

Recent proposals about the tubal origin of ovarian cancer challenged the traditional theory [11,19,20]. Large numbers of biomarkers have been used on the way to testify the new creation. PAX8 is a member of the PAX gene family, which includes nine well-characterized transcription factors (PAX1-9). Each member is directly implicated in the transcription of various genes, involved in organogenesis, morphogenesis, thyroid, renal and Müllerian cell differentiation [5]. This marker, initially identified in normal cells originating in Müllerian ducts, is also present in ovarian tumors and is characteristic for the epithelial phenotypes (serous, clear cell, and endometrioid) [5,21-23]. Moreover, PAX8 allows the differentiation between Müllerian and non-Müllerian origin in the case of an ovarian metastatic carcinoma that could derive from a primary tumor in pancreas, colon or mammary gland [24,25]. Due to its relatively specific for Müllerian epithelial cells, this marker is useful for the differentiation of the ovarian carcinomas, especially in the advanced stages, from breast carcinomas or malignant mesotheliomas exhibiting similar histologic features. Moreover, PAX8 has a diagnostic value as Müllerian differentiation marker in peritoneal effusions demonstrating the origin in high- and low-grade serous carcinomas [26].

In the present study, we examined the utility of PAX8 antibody combining with Calretinin in differential diagnosis of cancer cells within the ascites from patients with advanced “ovarian” cancer. All ascitic samples showing PAX8+/Calretinin- from patients who might receive NACT were 100% correlated to the final diagnosis of ovarian cancer. Even among the 20 cases with uncertain primary organ sites based on cytology diagnosis, 13 samples with PAX8+/Calretinin- were confirmed with ovarian cancer both immunohistochemically and clinicopathologically. The rest of the samples with PAX8-/Calretinin + or Calretinin- were not of ovarian origin. These results were further confirmed by examining the metastatic cancer and benign control groups. However, one thing we would like to emphasize here is that PAX8 can be also positive in renal cell carcinoma and benign Müllerian epithelia. This was demonstrated in one renal cancer case and 5 benign Müllerianosis samples. Fortunately, renal cell metastasis to ovary or clinically mimicking advanced ovarian cancer is extremely rare and benign Müllerian epithelia are easily identifiable under microscope. PAX8 staining can also be similarly applied to all peritoneal washing specimens in gynecologic malignancy when ovarian or Müllerian primay is questioned.

## Conclusion

In summary, PAX8 identifies all Müllerian derived benign or malignant epithelia. When combining with Calretinin, PAX8 is a sensitive and specific marker defining the cancers of ovarian or tubal origin. The application of PAX8 staining in appropriate clinical setting will be very useful for those patients presenting as advanced ovarian cancer prior to receiving neoadjuvant chemotherapy.

## Abbreviations

PAX8: Paired box 8; IHC: Immunohistochemistry; OEC: Ovarian epithelial cancer; NACT: Neoadjuvant chemotherapy; NCCN: The National Comprehensive Cancer Network.

## Competing interests

The authors declare that they have no competing interests.

## Authors’ contributions

WZ, BK, JMC designed the study, analyzed and interpreted the data. YW and YYW carried out experiments and data analysis. JL, ZY, BY and TZ contributed to the study analysis and interpretation of the data. All authors made substantial contributions to drafts of the manuscript and reviewed the final manuscript critically and authorized the submission. All authors read and approved the final manuscript.
